# Transverse Myelitis: A Case-Based Discussion of Infectious Etiologies

**DOI:** 10.7759/cureus.63079

**Published:** 2024-06-24

**Authors:** Divya Singh, Gavin deFisser, Sandra Samuel, Navreet T Chennu, Laura Polhemus, Wilson Rodriguez, Jafar Kafaie

**Affiliations:** 1 Neurology, Saint Louis University School of Medicine, St. Louis, USA; 2 Neurology, Saint Louis University Hospital, St. Louis, USA

**Keywords:** parainfectious, letm, streptococcus, meningitis, epstein-barr, transverse myelitis

## Abstract

Transverse myelitis (TM) is a frequently encountered inpatient neurological condition, usually with a broad differential of etiologies narrowed down by detailed history, temporal profile of symptom evolution, and pertinent diagnostic studies. We report a rare case of a 39-year-old man who presented with subacute onset of headaches and confusion, and three days later developed quadriplegia and areflexia. He was diagnosed with acute longitudinally extensive transverse myelitis (LETM) related to Epstein-Barr virus (EBV) superimposed on an initial presentation of streptococcal meningitis. As both etiologies are under-reported, we compare our case to the few similar cases in the literature to guide discussion of the clinical and radiologic findings of parainfectious TM related to EBV and streptococcal meningitis. Readers will have the challenge of attributing our patient’s myelitis to one of these parainfectious sources and are encouraged to evaluate for rare infectious etiologies in acute settings.

## Introduction

Acute transverse myelitis (TM) is an inflammatory disorder of the spinal cord characterized by rapidly progressive weakness, sensory deficits, and potentially bowel and bladder deficits. The etiologies of inflammation include post-infectious, systemic inflammation, multifocal central nervous system (CNS) disease, and idiopathic causes. While TM may occur at any level of the spinal cord, the most commonly affected area is at the thoracic level, usually causing bilateral weakness and sensory loss below the level of the lesion with either full or partial involvement of the motor and sensory fibers. The chances of full recovery without any deficits, recovery to a moderate degree of disability, and permanent disability are 33% respectively [1].

The most common causes of post-infectious myelitis include enteroviruses, West Nile virus, measles, syphilis, herpes simplex viruses, and neuroborreliosis (Lyme disease), although more rare infectious etiologies have been reported [1]. Epstein-Barr virus (EBV) is a double-stranded DNA herpesvirus commonly associated with infectious mononucleosis with symptoms of fever, lymphadenopathy, and tonsillar pharyngitis. Neurologic manifestations of EBV include encephalitis, meningitis, myelitis, and other neuropathies seen more frequently in immunosuppressed individuals than immunocompetent ones [2]. Direct infection of the CNS, systemic inflammation, multifocal nervous system diseases, and idiopathic causes [3] with either infiltration of lymphocytes or via molecular mimicry [4] produce aberrant immune reactions possibly responsible for the pathophysiology of TM. Here, we report a rare case of acute partial longitudinally extensive transverse myelitis (LETM) related to EBV in an immunocompetent patient, superimposed on an initial presentation of streptococcal meningitis.

## Case presentation

A 39-year-old male presented to the emergency department with a sore throat, ear pain, and a one-day history of headache, generalized weakness, and altered mentation. He was emergently intubated for a decreased level of consciousness and not being able to protect his airway. Computed tomography (CT) head was unremarkable. Initial lumbar puncture (LP) on the day of admission demonstrated severe hypoglycorrhachia (<5 mg/dL; normal 40-70 mg/dL), elevated protein (791 mg/dL; normal 15-40 mg/dL), and neutrophilic pleocytosis (WBC count of 2900; 98% neutrophils). Blood and cerebrospinal fluid (CSF) cultures were positive for *Streptococcus pneumoniae*. A two-week course of intravenous (IV) ceftriaxone 2g daily was initiated for pneumococcal meningitis and bacteremia. Additional CSF meningitis/encephalitis panels returned negative for enterovirus, herpes simplex virus 1 and 2, human herpesvirus 6, human parechovirus, varicella-zoster virus, and West Nile virus IgM. HIV serology, venereal disease research laboratory (VDRL), and aquaporin-4 IgG also returned negative.

**Figure 1:**
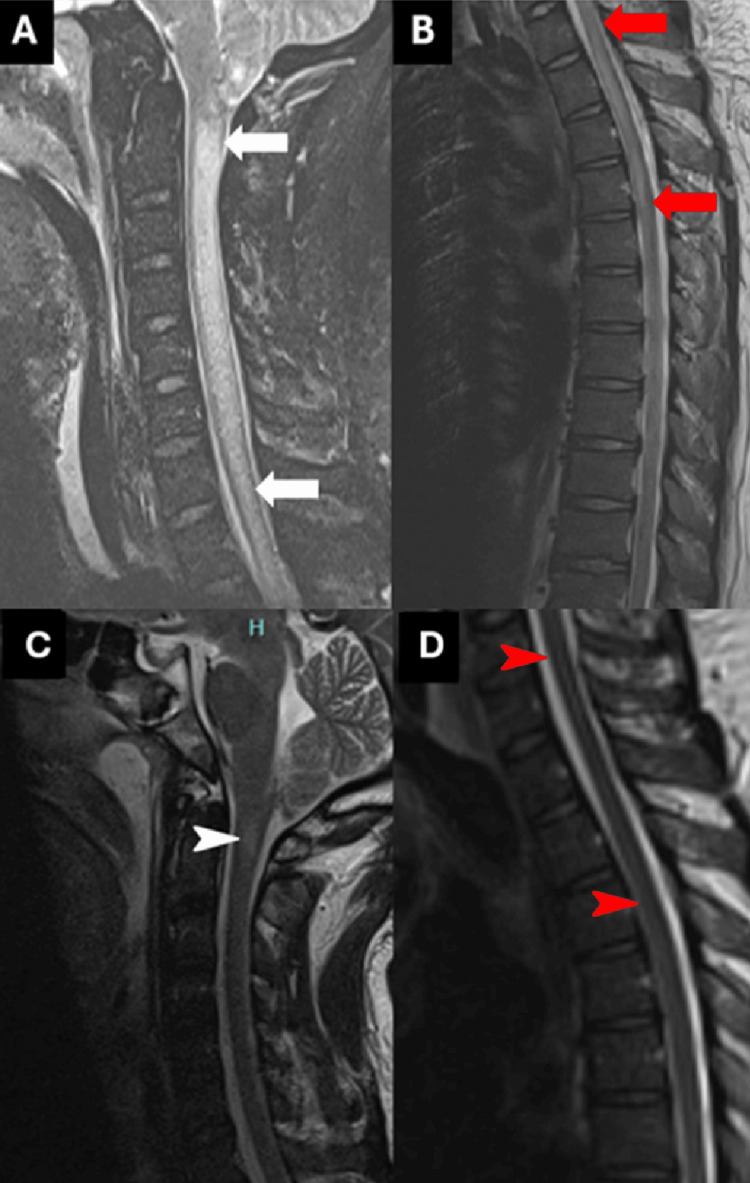
Initial sagittal T2 sequence of cervical and thoracic spine MRI showing multilevel intramedullary hyperintensity seen throughout the cervical spine (A, white arrows) and involving numerous segments of the upper thoracic spine (B, red arrows). Sagittal T2 sequence of cervical and thoracic spine MRI obtained three weeks after admission showing subtle hyperintensity at C1 level (C, white arrowhead), and resolution of intramedullary hyperintensity seen in upper thoracic spine (D, red arrowhead).

On the third day of hospitalization, his condition worsened with urinary retention, flaccid quadriplegia, and areflexia with intact sensation to pain, light touch, vibration, and proprioception throughout. Spinal magnetic resonance imaging (MRI) revealed diffuse longitudinal multilevel intramedullary T2 hyperintensities throughout the cervical spinal cord with extension to the upper thoracic spine without evidence of vascular insult (Figures 1A-1B). Clinically as well, the patient continued to be febrile. Repeat LP had CSF studies positive for EBV polymerase chain reaction (PCR); serum EBV IgG positive but negative IgM. IV ganciclovir was then initiated to complete a two-week course with fever resolution a couple of days after initiation. During this time, he also received methylprednisolone 1000 mg IV daily for five days followed by 60 mg prednisone daily (with continued tapering by 10 mg per week), and five plasmapheresis sessions with minimal clinical improvement.

Two weeks later, the patient underwent tracheostomy and percutaneous endoscopic gastrostomy (PEG) tube placement. Repeat spinal MRI three weeks after admission demonstrated significant interval resolution of intramedullary T2 hyperintensities and edema throughout the cervical and upper thoracic spinal cord (Figures 1C-1D). Clinically, encephalopathy resolved; however, there was no improvement in motor or sensory function. The patient was eventually discharged to a long-term acute care facility. At the eight-week follow-up, the patient was decannulated, had his PEG tube removed, and was able to walk with the assistance of a walker.

## Discussion

TM is an inflammatory myelopathy driven primarily by immunologic mechanisms affecting the spinal cord. It can have sensory, motor, or autonomic dysfunction with evidence of non-compressive T2 signal changes on MRI. When it spans ≥3 vertebral levels, it is further categorized as LETM. Etiologies of TM can be broadly separated into demyelinating, autoimmune, paraneoplastic, parainfectious, toxic-metabolic, and idiopathic [5]. Parainfectious sources of TM are vast. While viruses such as enterovirus and herpes simplex virus are well known [1], less common viral and bacterial etiologies cannot be disregarded.

Parainfectious EBV-induced TM is rare but not unprecedented. Several case reports have been published describing TM correlated with PCR-positive EBV in CSF [2,6-8]. However, in many of these, CSF studies were characteristic of viral infection (lymphocytic pleocytosis, normal glucose) [2,6,7], which is not comparable to our patient. The patients also had complete recovery within 10 days to four weeks after treatment with either high-dose steroids alone [7] or with intravenous immunoglobulin (IVIG) [6], acyclovir [2], or ganciclovir [8], while our patient continued to be bed-bound and quadriplegic upon discharge.

One challenge to our case is that TM as sequelae of streptococcal meningitis cannot be excluded. Two case reports have studied patients who presented after treatment for streptococcal meningitis with lower extremity weakness that evolved into paraplegia, both of whom made a slow recovery to a new baseline [9,10]. In a separate case series, Kastenbauer et al. described three patients who developed TM after bacterial meningitis. All cases developed persistent weakness and autonomic dysfunction of the bowel and bladder [11]. These poorer outcomes reflect more the clinical course that our patient experienced, pending long-term follow-up.

Our patient's initial presentation and CSF results reflected a clinical picture of acute bacterial meningitis without autonomic, motor, or sensory symptoms. It is possible that direct irritation from pneumococcal meningitis or a dysimmune reaction triggered by the pathogen caused TM, but nevertheless, viral reactivation in the setting of sepsis is also a known epiphenomenon. While we do not have evidence of a prior EBV infection in our case which is perhaps a limitation, EBV reactivation cannot be negated given positive CSF PCR. We suspect that it worsened the disease severity and contributed to new symptoms [12]. The evidence of radiological improvement with interval T2 hyperintensity resolution following high-dose steroids, plasma exchange, and ganciclovir favors EBV diagnosis. Perhaps our patient is most comparable to the case reported by Ngyuen et al. of a pregnant woman with EBV reactivation treated with steroids and IVIG who had radiographic resolution after treatment [6], aside from the fact that our patient did not clinically improve in the short term. Ultimately, it is difficult to say which parainfectious source caused our particular patient’s TM.

## Conclusions

Our case highlights a cause-and-effect dilemma often encountered in medicine. It is one of the very few cases reported in the literature that provides evidence of both EBV and *S. pneumoniae* as possible etiologies for TM in an immunocompetent young individual and illustrates an account of management and treatment strategies used. Furthermore, it highlights the epiphenomenon of viral reactivation in sepsis that is not frequently investigated in clinical settings. In conclusion, we emphasize for situations with such uncertainties to refrain from framing bias, as this may lead to fixation on one source and cause a delay in diagnostic studies and thus treatment of a potentially disabling neurological condition such as TM.
